# Clevidipine for the management of hypertension in the perioperative cardiac and noncardiac surgical settings: a systematic review

**DOI:** 10.3389/fmed.2025.1562681

**Published:** 2025-05-07

**Authors:** Alice Bottussi, Jacopo D’Andria Ursoleo, Viviana Teresa Agosta, Enrica Piazza, Edoardo Mongardini, Fabrizio Monaco

**Affiliations:** Department of Anesthesia and Intensive Care, IRCCS San Raffaele Scientific Institute, Milan, Italy

**Keywords:** antihypertensive drugs, cardiac surgery, clevidipine, noncardiac surgery, perioperative hypertension, perioperative medicine

## Abstract

**Introduction:**

Perioperative hypertension is a common occurrence in anesthetic practice, potentially leading to cardiac complications resulting in unfavorable patient outcomes. Clevidipine emerges in the current drug scenario as an antihypertensive agent with advantageous properties like ultra-short half-life, titratable effect, and ease of use.

**Methods:**

A systematic search of PubMed/MEDLINE, EMBASE, Cochrane Central Register of Controlled Trials and Google Scholar databases was performed aided by a specific search string, to provide a synthesis of the available body of literature regarding clevidipine administration for the management of hypertension in the perioperative cardiac and noncardiac surgical settings.

**Results:**

Eighteen documents (summarizing data from 2,066 patients) published between 1997 and 2023 were included in the present review. Clevidipine was extensively compared to both placebo and other commonly used antihypertensive medications.

**Discussion:**

Clevidipine demonstrated favorable efficacy and promising safety profiles. Moreover, it may represent a promising aid for the intraoperative management of hypertension, and a valuable addition to traditional antihypertensive drugs. However, significant gaps in research still persist, and further studies are warranted to better dissect its effects in frail populations and patients of different ethnicities.

**Systematic review protocol:**

PROSPERO (CRD42024568584).

## Graphical abstract

Schematic representation of the main article structure, objective, research methodology, and results. 
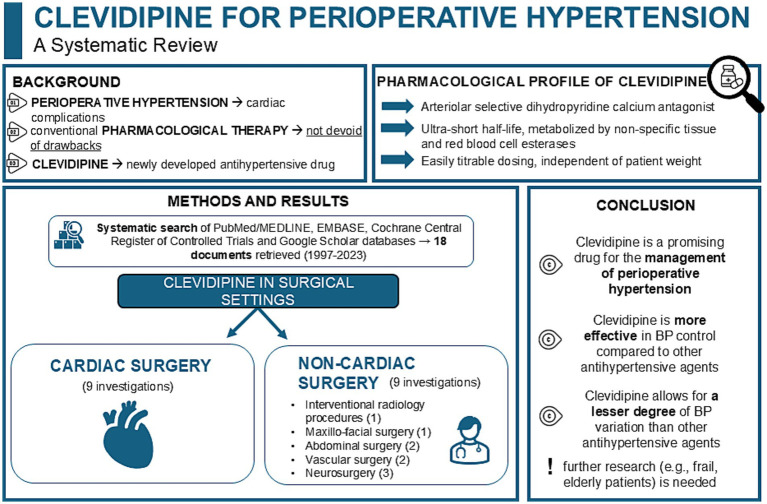

## 1 Introduction

Sudden increases in blood pressure represent a common occurrence in daily anesthetic practice and pose potential challenges in the safe management of patients undergoing surgery (1). As such, the occurrence of perioperative hypertension—defined as an increase in blood pressure greater than 20% compared to its basal values—is often associated with acute, pain-induced sympathetic stimulation, hypothermia, hypoxia, intravascular volume overload, and/or preoperative discontinuation of long-standing antihypertensive medications (1).

Interestingly, the concept of ‘safe’ perioperative care for patients involves both the prevention and timely, appropriate management of hypertensive episodes. This also implies avoiding pharmacologic overtreatment and preventing intraoperative hypotension, as both hypertension and hypotension have been linked to increased perioperative mortality (2). Moreover, evidence inferred from the published literature also postulates an association between intraoperative fluctuations in mean arterial pressure (MAP) and the occurrence of perioperative cardiac complications (e.g., myocardial ischemia) alongside adverse outcomes (e.g., increased 30-day mortality) (2, 3).

Therefore, optimal management of hypertension in the perioperative setting is pivotal to ensure better patient postoperative outcomes. In this regard, while the administration of ‘conventional’ pharmacotherapies (e.g., esmolol, nicardipine, nitroglycerine, urapidil, and sodium nitroprusside [SNP]) proved to be effective in managing abrupt MAP fluctuations, these drugs are not devoid of risks (1). For instance, esmolol is contraindicated in patients already receiving β-blocker therapy, in case of pre-existing bradycardia, in instances of decompensated heart failure and it can also precipitate bronchospasm. Furthermore, while SNP was proved to decrease cerebral blood flow while rising intracranial pressure, it also potentially leads to accumulation of cyanide thereby causing cytotoxicity via the release of nitric oxide (1). Additionally, nitroglycerin is not recommended as first-line therapy for hypertensive emergencies/urgencies, yet it may constitute a valuable adjunct to other antihypertensive agents, particularly in the setting of hypertension associated with acute coronary syndromes or acute pulmonary edema (4). Within this consolidated panorama, clevidipine is emerging as a possible new therapeutic aid in the management of acute perioperative hypertension.

Clevidipine is an arteriolar-selective, ultra-short acting dihydropyridine calcium antagonist, approved in August 2008 by the Food and Drug Administration (FDA) (5). It exerts its therapeutic effects by inhibiting L-type calcium channels in a voltage-dependent manner, thereby selectively reducing the muscular tone of small arteries, and thus reducing peripheral vascular resistance without causing variations in either venous capacitance vessels or cardiac filling pressures. Its metabolism—which is based on non-specific tissue and red blood cell esterases—is completely independent from both the patient renal and hepatic function, thus making this drug suitable even in patients with impairment of their renal and/or hepatic function. Moreover, clevidipine is characterized by an ultra-short half-life thanks to its plasmatic metabolism. Its fast onset and offset, alongside its dosing properties—which is easily titratable and independent of patient weight—contribute to the ease-of-use and appeal of this novel drug (6, 7).

Given the promising pharmacokinetic/pharmacodynamic profile of clevidipine and its ever-increasing use as part of the daily perioperative hemodynamic management in patients experiencing perioperative hypertension, we conducted a systematic review to retrieve and summarize all the available evidence regarding its use in both the cardiac and noncardiac surgery settings. By reporting on the key features, indications, outcomes and adverse events emerging from the current body of literature, we aim to equip the practicing anesthesiologist with insightful perspectives to implement clevidipine as a part of the daily management of perioperative hypertension involving patients across several surgical settings (Graphical abstract).

## 2 Materials and methods

### 2.1 Search strategy

In line with the guidelines from the Cochrane Collaboration and Centre for Reviews and Dissemination, we performed a systematic literature review according to the Preferred Reporting Items for Systematic Reviews and Meta-Analyses (PRISMA) checklist (Supplementary Table S1) and registered the study protocol in the prospective international register of systematic reviews (PROSPERO) with the unique registration number CRD42024568584.

A comprehensive search on PubMed/MEDLINE, EMBASE, the Cochrane Central Register of Controlled Trials and Google Scholar databases was carried out by two trained and independent investigators (A.B. and E.P.) aiming to identify pertinent studies (up to August 31, 2024, with no inception limits).

Search terms including “clevidipine,” “clevidipine butyrate” alongside its trade name “Cleviprex®” were incorporated within the search string. Keywords and free terms were combined with the aid of the Boolean operator ‘OR’ to enhance search precision. Further information regarding the search strategy is made available in Supplementary material, Search string.

The EndNote X9 (Clarivate Analytics) was used to remove duplicate publications and screening of the resulting citations was performed following upload to Rayyan (8).

Both backward and forward snowballing of the references of selected articles were performed to identify potential additional studies suitable for possible inclusion into the present systematic review. Only articles written in English were considered for potential inclusion.

### 2.2 Study selection

Two independent investigators (A.B. and E.M.) evaluated each of the references obtained from the database search of the published literature independently at both title and abstract levels.

Full-text articles were consulted in cases where concerns or disagreements occurred, and any disagreements were resolved through discussion involving a third, senior investigator (J.D.U.).

#### 2.2.1 Inclusion criteria

Studies of any design (including case reports or series) published in peer-reviewed journals and written in English reporting on original experience of intravenous (iv) clevidipine administration either alone or in combination with other antihypertensive drugs for the management of perioperative hypertension (as defined by study Authors) in patients aged 16 years or older scheduled to undergo any kind of surgical procedures (in both the cardiac and noncardiac settings, either elective or emergent) were identified and carefully assessed.

#### 2.2.2 Exclusion criteria

Studies reporting data from the pediatric population, publications lacking original data (including reviews, systematic reviews, meta-analyses, commentaries, conference abstracts, letters, and editorials), studies with overlapping populations or performed in animal models and works published in languages other than English were excluded from this review.

No additional limitations on study design were applied.

### 2.3 Data extraction and study characteristics

The PICO (Patient/Population/Problem, Intervention, Comparison/Control, Outcome) approach together with standardized forms were used to carry out data extraction. Specifically, the adult population undergoing any kind of surgical procedures (in both the cardiac and noncardiac settings, either elective or emergent) who developed perioperative hypertension (as defined by study Authors) was considered as the patient group (P). Interventions involving the intravenous infusion of clevidipine—either alone or in combination with other drugs (I)—versus any comparators (C) when and if present, for blood pressure management (as defined by study Authors) were assessed (O).

Extracted information included details on each of the original investigations retrieved (i.e., first author, publication year, study design, total number of patients involved, number of patients in the intervention group, number of patients in the control group, surgical setting in which the study drug was administered, study drug administration route, study drug infusion rate, control drug [when and if present] administration route, control drug [when and if present] infusion rate, timing of study drug initiation, blood pressure target [as defined by study Authors)] study drug adverse event(s), control drug adverse event(s) and patient outcome data.

A summary of the key features, indications, outcomes and adverse events emerging from the studies performed in the cardiac surgery setting are presented in Table 1.

**Table 1:** Characteristics, outcomes, and adverse events of the retrieved studies investigating the role of clevidipine in cardiac surgery.

First author, year	Study design	CLV group (number of pts)	Comparator group (number of pts)	CLV infusion rate	Comparator infusion rate (if any)	CLV timing	Target blood pressure (mmHg)	Primary outcome	CLV adverse events (if any)	Comparator adverse events (if any)	Key features
Aronson, 2008 (11)	RCT	1st: 268 2nd: 296 3rd: 188	NTG: 278 SNP: 283 NIC: 193	Min: 0.4 mcg·kg^−1^·min^−1^ Max: 8 mcg·kg^−1^·min^−1^	NTG median: 11.3 mL·h^−1^ SNP median: 8.5 mL·h^−1^ NIC median: 33.6 mL·h^−1^	NTG: Perioperative SNP: Perioperative NIC: Postoperative	*Intraoperatively*: SAP: 65–135 *Pre- and postoperatively*: SAP 75–145	Safety of CLV in the treatment of hypertension perioperatively, assessed by the incidences of death, stroke, MI and renal dysfunction	*Primary outcome* Death: 2.8% MI: 2.3% Stroke: 1.1% Renal dysfunction: 7.9% *SAEs* AF: 2.4% Respiratory failure: 1.1% Acute renal failure: 2.3% VF: 0.9% Cardiac arrest: 0.5% CVA: 0.5% Hemorrhage: 0.5%	*Primary outcome* Death: 3.8% MI: 2.4% Stroke: 1.71% Renal dysfunction: 7.9% *SAEs* AF: 2.4% Respiratory failure: 2.5% Acute renal failure: 1.7% VF: 1.5% Cardiac arrest: 1.1% CVA: 1.1% Hemorrhage 1.1%	*Primary outcome*: until 30 days postoperatively Death (*p* = 0.26), stroke (*p* = 0.38), MI (*p* = 0.88), renal dysfunction (*p* = 0.99) *AEs*: AF: 33.6% vs. 32.0% (CLV vs. NTG); 36.1% vs. 32.2% (CLV vs. SNP); 35.6% vs. 35.2% (CLV vs. NIC), *p* = NS
Colomy, 2021 (17)	Observational retrospective study	29	NIC: 38	Min: 1 mg·h^−1^ Max: 21 mg·h^−1^	Min: 5 mg·h^−1^ Max: 15 mg·h^−1^	Intraoperative, Postoperative	SAP range: ± 30 (usually 110–140)	The percentage of time spent within patient specific SBP goal	Hypotension: 27.6% Vasopressor use: 3.5% SCr ↑ ≥ 0.3 mg/dL in 72 h: 27.6% Tachycardia: 10.3% AF: 0%	Hypotension: 23.7% Vasopressor use: 18.4% SCr ↑ ≥ 0.3 mg/dL in 72 h: 39.5% Tachycardia: 2.6% AF: 2.6%	*Primary outcome*: CLV median 55.2% NIC median 36.4% *AEs*: *p* > 0.05
Freiberger, 2016 (16)	Retrospective study	40	SNP: 40	Min: 1 mg·h^−1^ Max: 21 mg·h^−1^	Min: 0.25 mcg·kg^−1^·min^−1^ Max: 10 mcg·kg^−1^·min^−1^	Postoperative	n/a	Mean number of times the SBP rose above 140 mmHg	AF: 47.5% (*p* = 0.499) SCr ≥ 1.5 times admission baseline: 20% AST > 123 units/L: 5% ALT >162 units/L: 5% In-hospital mortality: 10%	AF: 40% SCr ≥ 1.5 times admission baseline: 12.5% AST > 123 units/L: 2.5% ALT >162 units/L: 2.5% In-hospital mortality: 0%	*Primary outcome*: CLV 22.7% SNP 12.6% *AEs*: AF (*p* = 0.499) SCr ≥ 1.5 times admission baseline (*p* = 0.363) AST > 123 units/L (*p* = 1.000) ALT >162 units/L (*p* = 1.000) In-hospital mortality (*p* = 0.116)
Kieler-Jensen, 2000 (5)	Cross-over study	13	SNP: 13	Mean: 2.27+/−0.65 mcg·kg^−1^·min^−1^	Mean (phase 1): 1.14+/−0.21 mcg·kg^−1^·min^−1^ Mean (phase 2): 0.68+/−0.04 mcg·kg^−1^·min^−1^	Postoperative	MAP 70–80	Effects of incremental doses of CLV on central hemodynamics, coronary blood flow and cardiac metabolism	n/a	n/a	*Phase 1*: SNP infusion CLV infusion *Phase 2*: SNP infusion CLV caused ↓ MAP, ↓ vascular resistances, no changes in myocardial O_2_ consumption
Levy, 2007 (13)	RCT	53	Placebo: 52	Min: 0.4 mcg·kg^−1^·min^−1^ Max: 8 mcg·kg^−1^·min^−1^	Min: 0.4 mcg·kg^−1^·min^−1^Max: 8 mcg·kg^−1^·min^−1^	Preoperative	↓ SAP (≥ 160) by at least 15%	↓ AP preoperatively and tolerance	↑ HR: median 71 bpm Pyrexia: 18.9% AF: 13.2% Acute renal failure: 9.4% Nausea 5.7% MI: 0% Death: 1.9%	↑ HR: median 76 bpm Pyrexia: 13.7% AF: 11.8% Acute renal failure: 2% Nausea 9.8% MI: 3.9% Death: 0%	*Efficacy results*: CLV 92.5% Placebo 17.3% *AEs*: until hospital discharge or 7 days *AEs*: no significant difference *Treatment-related AEs*: CLV pts. 9.4% Placebo pts. 3.9%
Merry, 2014 (15)	RCT	49	NTG: 51	Min: 0.2 mcg·kg^−1^·min^−1^ Max: 8 mcg·kg^−1^·min^−1^	Min: 0.4 mcg·kg^−1^·min^−1^Max: clinician-determined	Intraoperative	MAP within ± 5 of a predetermined target	AP control pre-CABG	Hypotension: 26.5% Confusional state: 4.1% AF: 2% Renal acute failure: 0%	Hypotension: 15.7% Confusional state: 0% AF: 9.8% Renal acute failure: 3.9%	Dose rates in excess of 4.4 mcg·kg^−1^·min^−1^ limited to a total of 120 min 5 pts. in each group stopped the medication due to an AE AEs reported in this table: possible CLV-related AEs
Patel, 2012 (10)	Case report	1	n/a	2–4 mg·h^−1^	n/a	Intraoperative, Postoperative	MAP 65–75	Prevention of vasospasm after CABG surgery	HR: mild ↑	n/a	*Primary outcome*: postoperatively no vasospasm or coronary ischemia
Singla, 2008 (14)	RCT	61	Placebo: 49	Min: 0.4 mcg·kg^−1^·min^−1^Max: 8 mcg·kg^−1^·min^−1^	Min: 0.4 mcg·kg^−1^·min^−1^ Max: mcg·kg^−1^·min^−1^	Postoperative	↓ SAP (≥ 140) by at least 15%	The incidence of treatment failure	*AEs* Nausea: 21.3% AF: 21.3% Insomnia:11.5% Edema: 8.2% Atelectasis: 3.3% *SAEs* Thrombophlebitis: 1.5% Pneumonia, respiratory failure, AF, hemorrhage: 3.3%	*AEs* Nausea: 12.2% AF: 12.2% Insomnia: 6.1% Edema: 12.2% Atelectasis: 10.2% *SAEs* Thrombophlebitis: 0% Pneumonia: 0% Respiratory failure: 2% AF: 2% Hemorrhage: 2%	*Primary outcome*: CLV 8.2% Placebo 79.6% *AEs*: until hospital discharge or 7 days postoperatively *AEs*: no significant difference CLV discontinued for safety reason in 3 pts.
Vuylsteke, 2000 (9)	Observational study	17	n/a	Min: 0.7 mcg·kg^−1^·min^−1^Mean: 1.3+/− 0.4 mcg·kg^−1^·min^−1^	n/a	Intraoperative	*Before CPB*: MAP 70–75 *During CPB*: MAP 55–60	Pharmacokinetics and the pulmonary extraction ratio	n/a	n/a	During CPB, clearance of CLV ↓ Max infusion rate: 22 μg kg^−1^ min^−1^ allowed

pts, patients; IMA, internal mammary artery; NTG, nitroglycerin; CLV, clevidipine; SNP, sodium nitroprusside; min, minimum; MAP, mean arterial pressure; CBP, cardiopulmonary bypass; max, maximum; SAP, systolic arterial pressure; AP, arterial pressure; HR, heart rate; AF, atrial fibrillation; MI, myocardial infarction; AEs, adverse events; SAEs, serious adverse events; NIC, nicardipine; VF, ventricular fibrillation; CVA, cerebrovascular accident; *p*, *p* value; NS, non-significant; CABG, coronary artery bypass graft surgery; SCr, serum creatinine.

Table 2 summarizes insights from the studies performed in the noncardiac surgery setting.

**Table 2:** Characteristics, outcomes, and adverse events of the retrieved studies investigating the role of clevidipine in noncardiac surgery.

First author, year	Study design	CLV group (number of pts)	Comparator group (number of pts)	CLV infusion rate	Comparator infusion rate (if any)	CLV timing	Target blood pressure (mmHg)	Primary outcome	CLV adverse events (if any)	Comparator adverse events (if any)	Key features
Abdominal surgery											
Lindstrom, 2016 (18)	Case report	2	n/a	Min: 4 mg·h^−1^, bolus 1 mg Max: 12 mg·h^−1^	n/a	Intraoperative	n/a	Treatment of hypertension caused by pheochromocytoma with CLV only	↑ HR: max 120 bpm	n/a	n/a
Luis-Garcìa, 2018 (19)	Case report	1	n/a	Min: 2 mg·h^−1^ Max: 8 mg·h^−1^	n/a	Intraoperative Postoperative	n/a	Treatment of hypertension caused by pheochromocytoma	n/a	n/a	*Preoperatively*: Nifedipine 20 mg/12 h, Atenolol 50 mg/24 h *Occurred events*: none
Maxillo-facial surgery											
Kline, 2010 (20)	Case report	1	n/a	1 mg·min^−1^ 5 mg·min^−1^	n/a	Intraoperative	n/a	Treatment of refractory hypertension caused by undiagnosed pheochromocytoma	n/a	n/a	*Drugs used before CLV*: Labetalol 20 mg Hydralazine 20 mg NTG bolus 20–100 μg Phentolamine 5 mg
Interventional radiology procedures											
Meyer, 2009 (21)	Case report	1	n/a	Min: 2 mg/h Max: 8 mg/h	n/a	Intraoperative, Postoperative	SAP 120	AP control during anesthetic care	↑ HR: from 72 to 80 bpm	n/a	Target pressure achieved in 100% of pts. *Occurred events*: none
	Case report	1	n/a	Min: 1 mg/h Max: 2 mg/h	n/a	Intraoperative, Postoperative	SAP 110–120	AP control during anesthetic care	↑ HR: from 80–90 to 90–94 bpm	n/a	Target pressure achieved in 100% of pts. *Occurred events*: none
	Case report	1	n/a	Min: 2 mg/h Max: 3 mg/h	n/a	Intraoperative, Postoperative	SAP 100–120	AP control during anesthetic care	n/a	n/a	Target pressure achieved in 100% of pts. *Occurred event*: aneurysm bleeding
Neurosurgery											
Bekker, 2010 (22)	Clinical report	21	n/a	Min: 10 mg/h Max: 55 mg/h	n/a	Intraoperative, Postoperative	SAP < 130	Patients in whom SAP could be controlled with CLV only	Transient hypotension (≤5 min): 2 episodes	n/a	*Primary outcome*: 81% *Transient hypotension* (≤5 min): 20 episodes non-CLV-related
Borrell-Vega, 2020 (23)	Observational study	12	NIC: (same) 12	n/a	Max 15 mg/h	Perioperative	SAP <140 for ICH SAP <160 for SAH SAP <180 for IS	Comparison of the median % of time spent at targeted SAP goals during NIC and CLV administration	Hypotension: 1 pt. Median % of time spent with HR > 100: 2.2%	Hypotension: 1 pt. Median % of time spent with HR > 100: 13.1%	*Primary outcome*: CLV 93.4% NIC 76.2% *Median % of time spent with HR > 100*: *p* = 0.250
Short, 2020 (24)	Case report	1	n/a	Min: 10 mg/h Max: 16 mg/h	n/a	Postoperative	MAP 60–80	CLV-induced hypoxemia	Refractory hypoxemia	n/a	CLV was discontinued and hypoxemia resolved within 1 h
Vascular surgery											
Haurax, 1997 (25)	Observational preclinical study	IMA segments from 6 pts	NTG: IMA segments from 6 pts.	n/a	n/a	n/a	n/a	Effects of CLV on human IMA precontracted in the presence or absence of endothelium and comparison with those of NTG	n/a	n/a	CLV caused effectively vasodilatation both in presence or absence of endothelium
Pascual, 2017 (26)	Correspondence	1	n/a	Min: 2 mg·h^−1^ Max: 6 mg·h^−1^	n/a	Intraoperative	MAP < 90	PSA after a SKPT: the efficacy and safety of CLV in the AP control	none	n/a	After stopping CLV infusion: Nifedipine 60 mg, Bisoprolol 5 mg

pts, patients; min, minimum; max, maximum; SAP, systolic arterial pressure; CLV, clevidipine; MAP, mean arterial pressure; NIC, nicardipine; IHC, intracranial hemorrhage; SAH, subarachnoid hemorrhage; IS, ischemic stroke; HR, heart rate; *p*, *p* value; NTG, nitroglycerine; PSA, pseudoaneurysm; SKPT, simultaneous kidney-pancreatic transplant.

### 2.4 Risk of bias quality assessment

The Risk Of Bias In Non-Randomized Studies-of Interventions (ROBINS-I) tool for observational studies and the Risk-of-Bias tool for randomized trials (RoB2) by Cochrane^1^ were employed to perform the risk of bias assessment of retrieved investigations, as reported in Supplementary Tables S2, S3. During the review process, disagreements were resolved by consensus or by discussion with an additional investigator. The evaluation method classified risk levels into three tiers: “high risk of bias,” “some concerns,” or “low risk of bias.” For an investigation to be classified as “low risk of bias,” it was necessary of all areas to be assessed as low risk of bias.

### 2.5 Statistical analysis

The results from individual studies were presented—typically encompassing predictive performance for predefined outcomes—in a narrative fashion so as to elucidate its relevance with respect to the research objectives of the systematic review itself. Of note, the heterogeneity in the literature prevented us from conducting formal data synthesis or analysis.

## 3 Results

**Figure 1:**
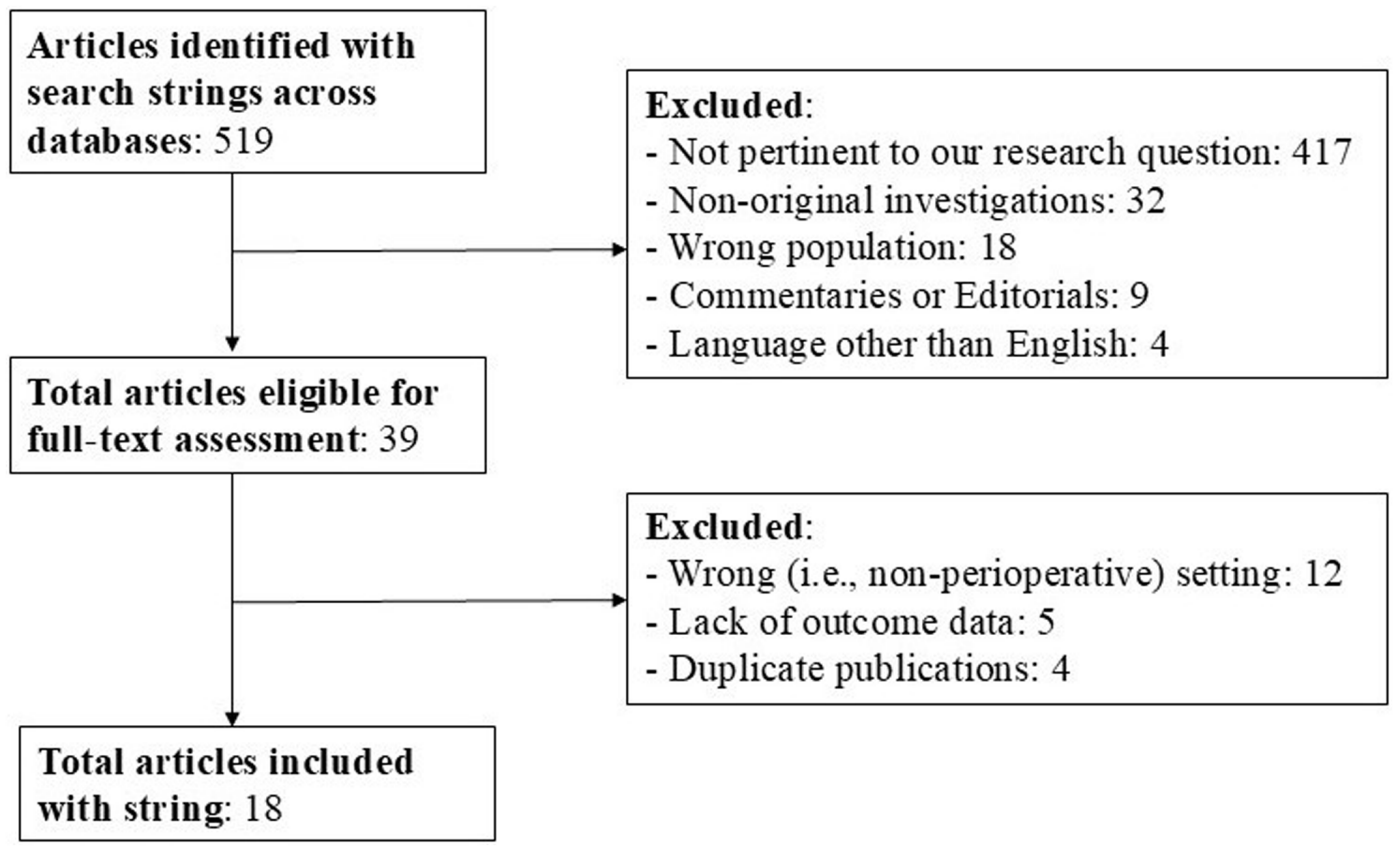
PRISMA flow-chart. Flow-chart of the studies selection process.

Our search strategy conducted on the PubMed/MEDLINE, EMBASE, the Cochrane Central Register of Controlled Trials and Google Scholar databases yielded a total of 519 documents (from inception until August 31, 2024). Figure 1 summarizes the process of studies identification, screening and inclusion. A total of 501 documents were irrelevant to the research topic and were excluded. Consequently, 18 documents were retrieved and included in the present systematic review following grouping in the categories as below. All of the retrieved studies were published between 1997 and 2023, involved a total of 2,066 patients and investigated the administration of clevidipine in the perioperative settings of cardiac surgery (9 studies, including a total of 2,019 patients) and noncardiac surgery (9 studies, including a total of 47 patients), the latter further spanning the sub-specialties of abdominal, maxillo-facial, vascular surgery, interventional radiology procedures, and neurosurgery.

### 3.1 Cardiac surgery

Between 2000 and 2023, a total of 9 studies (50% of the total number of retrieved publications) investigating the role of clevidipine in the setting of cardiac surgery were published. The majority of these (*n* = 5) were conducted in the United States, while two took place in Europe and one in New Zealand.

The studies by Kieler-Jensen et al. and Vuylsteke et al. were both published in 2000 and reported on the pharmacokinetics and pharmacodynamics of clevidipine. Specifically, Kieler-Jensen et al. investigated the hemodynamic effects of clevidipine on coronary and systemic vessels. In their study, they identified two phases: in phase 1 (13 patients), patients were hypertensive (i.e., required vasodilatory treatment with sodium nitroprusside [SNP] to maintain a MAP of 70–80 mmHg) and the authors dissected on the efficacy of clevidipine versus SNP to control blood pressure using a cross-over design; in phase 2 (9 patients), the patients were normotensive (defined as a systolic blood pressure [SBP] > 140 mmHg) and the authors elucidated on the hemodynamic and cardiac metabolic effects of incremental infusion rates of clevidipine (5). In phase 1, when comparing the clevidipine infusion to the SNP infusion, the authors observed: (i) lower values of systemic vascular resistance (SVR) and heart rate (HR); (ii) higher values of preload, stroke volume (SV) and pulmonary vascular resistance (PVR); (iii) no differences in myocardial lactate metabolism or oxygen extraction. In phase 2, clevidipine infusion induced a dose-dependent decrease in MAP, SVR and PVR, an increase in SV, with no changes in the preload or in HR. Myocardial oxygen extraction decreased from 54 to 45%, due to direct coronary vasodilation. The blood clearance was estimated at 0.05 L·min^−1^·kg^−1^, the volume of distribution at the steady state was 0.08 L·kg^−1^ and the initial and terminal half-lives were <1 min and 4 min, respectively (5). Vuylsteke et al. enrolled 17 patients to receive clevidipine as an iv infusion before cardiopulmonary bypass (CPB), with 8 patients receiving clevidipine also during hypothermic CPB. The authors subsequently performed a pharmacokinetic analysis—alongside the calculation of pulmonary extraction ratio on mixed venous and arterial blood samples. To describe the pharmacokinetic profile of clevidipine, a two-compartment model with zero-order input was used, both before and during CPB. The authors observed virtually-identical concentrations of clevidipine in mixed venous and arterial blood, thus suggesting a negligible pulmonary metabolism of the drug. A marked reduction in the total blood clearance of clevidipine was observed during CPB (0.03 L·min^−1^·kg^−1^ during bypass versus 0.055 L·min^−1^·kg^−1^) and explained as a consequence of reduced patient body temperature (9).

The role of clevidipine in the prevention of vasospasm after radial and internal mammary artery (IMA) grafts during coronary artery bypass grafting (CABG) was investigated by Patel et al. as well. In their case report, a 67-year-old woman underwent urgent CABG surgery. General anesthesia induction was obtained with etomidate (20 mg), fentanyl (500 mcg) and rocuronium (50 mg); isoflurane (expired concentration 0.5–1%) was titrated during the surgery to maintain the bispectral index (BIS) in the 40–60 range. During the harvesting of the radial artery and IMA, a clevidipine infusion was initiated at 2 mg·h^−1^. Five minutes after the start of clevidipine infusion, HR increased from 62 to 78 bpm, while blood pressure decreased from 120/62 mmHg (MAP 80 mmHg) to 100/50 mmHg (MAP 64 mmHg). MAP was maintained at 65 to 75 mmHg by increasing the clevidipine infusion from 2 to 4 mg·h^−1^. During CBP, the clevidipine infusion was reduced to 2 mg·h^−1^ and then increased upon separation from CPB at 4 mg·h^−1^ to maintain a MAP of 60–75 mmHg with a HR of 80–90 bpm. Clevidipine infusion was continued for the first 12 h post-operatively at an infusion rate of 2 mg·h^−1^, after which the patient was transitioned to oral diltiazem, with no evidence of vasospasm or coronary ischemia (10).

In 2008, the ECLIPSE trial was published (11). The authors analyzed data from three prospective, randomized, open-label, parallel comparison studies of clevidipine versus nitroglycerine and versus SNP in the perioperative period, and versus nicardipine postoperatively in patients undergoing cardiac surgery. Of the 1964 patients enrolled, 1,512 met the inclusion criteria. Patients were randomized in a 1:1 ratio and the investigated outcomes were: incidence of death, myocardial infarction, stroke or renal dysfunction at 30 days, adequacy and precision of blood pressure control. The authors found no significant difference in mortality between the three groups (i.e., clevidipine, nitroglycerine, nicardipine), while mortality was significantly higher for patients treated with SNP. Clevidipine was more effective compared to nitroglycerine and SNP in maintaining blood pressure within a predefined range. Also, clevidipine was non-inferior to nicardipine in maintaining blood pressure within a predetermined range. However, when this range was narrowed, clevidipine granted a better blood pressure control, with fewer excursions outside of the desired range compared with nicardipine. These results were confirmed by a subsequent safety analysis of the ECLIPSE trials published in 2009, where clevidipine was found to be as safe as nicardipine, nitroglycerin and SNP for the treatment of perioperative hypertension in cardiac surgery patients (12). Moreover, clevidipine provided better blood pressure control within the first 24 h, when compared with nitroglycerin and SNP. Furthermore, patients treated with SNP exhibited more frequently SBP values below the target range than patients treated with clevidipine. The need for adjunctive antihypertensive agents was similar between the groups. Lastly, a relevant finding from all the studies was that blood pressure results following treatment with clevidipine were remarkably similar, implying the predictable effects of clevidipine on blood pressure throughout the perioperative course.

Similar results regarding the efficacy and safety of clevidipine can be derived from a study by Levy et al., in which the authors investigated the antihypertensive efficacy of clevidipine versus placebo in the preoperatory cardiac surgery setting in a randomized, double-blind, multicenter trial (13). One hundred and five patients met the inclusion criteria (i.e., SBP > 160 mmHg) and were randomized to receive either a clevidipine infusion (0.4–0.8 mcg·kg^−1^·min^−1^) or placebo (20% lipid emulsion) for at least 30 min. Treatment failure was defined as a failure to reduce SBP by more than 15% from baseline or discontinuation of treatment for any reason. Patients treated with clevidipine showed a high rate (92.5%) of treatment success and a significantly lower rate (4 out of 53, 7.5%) of treatment failure than patients receiving placebo (43 out of 52, 82.7%). Target blood pressure was achieved at a median of 6 min (95% confidence interval: 6–8 min) with clevidipine. A modest increase in HR was observed in patients treated with clevidipine. Adverse events (i.e., pyrexia, atrial fibrillation, acute renal failure, nausea) were similar in both groups.

The following year, the ESCAPE-2 study by Singla et al. was published—a randomized, double-blind, placebo-controlled trial exploring the efficacy of clevidipine versus placebo in the post-operatory cardiac surgery setting (14). One hundred ten patients met the inclusion criteria (SBP > 140 mmHg within 4 h of admission to a postoperative setting, needing SBP reduction of at least 15%) and received either an infusion of clevidipine (0.4–0.8 mcg·kg^−1^·min^−1^) or placebo (20% lipid emulsion) for 30 min to 1 h (unless treatment failure occurred sooner). The primary endpoint was treatment failure, defined as the inability to decrease SBP of at least 15% or the occurrence of treatment discontinuation within the first 30 min. Clevidipine showed a significant lower incidence of treatment failure (5 of 61, 8.2%) with respect to placebo (39 of 49, 79.6%). Treatment success was obtained in 91.8% of clevidipine-treated patients, with a median time to achieve target SBP of 5.3 min (95% confidence interval: 4–7 min). Adverse events (i.e., nausea, atrial fibrillation, insomnia, edema, atelectasis) rate was similar in both groups (14).

In more recent years, the efficacy of clevidipine compared to antihypertensive drugs traditionally administered in cardiac surgery has also been extensively investigated. In 2014, Merry et al. compared clevidipine and nitroglycerin for blood pressure management in patients undergoing CABG in a randomized, multicenter, double-blind study (15). One hundred fourteen patients were enrolled and randomized to receive either clevidipine (0.2–0.8 mcg·kg^−1^·min^−1^) or nitroglycerin (0.4 mcg·kg^−1^·min^−1^ further titrated to a clinician-determined maximum dose) from anesthesia induction and up to 12 h post-operatively. The primary outcome was the efficacy of the drug in maintaining MAP within ±5 mmHg of a clinician-determined range, expressed as the area under the curve for the total time each patient’s MAP was outside the target MAP range from drug initiation to establishment of CPB, normalized per hour. A total of 49 patients received clevidipine (4 were then excluded from the analysis) and 51 received nitroglycerin (3 were then excluded from the analysis). Clevidipine met the predefined non-inferiority study criterion. The authors recorded no relevant differences in the incidence of myocardial ischemia, acute myocardial infarction, total blood loss, total fluid input, or total fluid output between the two groups. The incidence and type of adverse events (i.e., death, hypotension, ischemia, atrial fibrillation) were similar between the groups. Subsequently, clevidipine was compared to SNP in a single-center, retrospective, cohort study including patients treated for postoperative SBP control by Freiberger et al. The authors defined efficacy as the mean number of times the SBP rose above 140 mmHg and considered as secondary outcomes a comparative cost and safety analysis, with 40 patients being enrolled in each arm. In the clevidipine group, the authors found a higher incidence of SBP values >140 mmHg. However, no differences in safety outcomes, nor in the number of patients who received as-needed antihypertensive, nor in mean number of as-needed antihypertensive were recorded. The authors instead found a difference in infusion duration (longer in the clevidipine group), number of infusions dispended (greater in the clevidipine group), length of hospital stay (longer in the clevidipine group). However, clevidipine was less expensive than SNP at the time of the review (16). Lastly, Colomy et al. compared clevidipine to nicardipine in a single-center, retrospective, comparative study. Sixty seven patients met the inclusion criteria and received either clevidipine (29 patients) or nicardipine (38 patients) to control perioperative hypertension in the cardiac surgery setting. The outcomes investigated were: (i) the percentage of time spent within patient-specific blood pressure target, (ii) incidence of hypertensive events per patient, (iii) safety outcomes and cost of treatment. The median percentage of time spent within the predetermined blood pressure range was higher in clevidipine-treated patients (55.2% versus 36.4% for nicardipine); however, the authors did not find differences in safety outcomes (i.e., vasopressor use, serum creatinine elevation, new-onset tachycardia and/or atrial fibrillation), although the cost of treatment was higher for clevidipine (17).

### 3.2 Noncardiac surgery

Although studies investigating the role of clevidipine in noncardiac surgical settings account for approximately half of the included studies, the total number of patients (*n* = 47 across 9 studies) remains very low. Moreover, the available literature is predominantly composed of case reports or case series, constraining the generalizability of the presented evidence regarding the role of clevidipine for perioperative hypertension in noncardiac surgery.

#### 3.2.1 Abdominal surgery

Two studies elucidating on the use of clevidipine in the setting of abdominal surgery were retrieved. Both were case reports published between 2010 and 2018. In the setting of abdominal surgery, a particular need for careful blood pressure control arises in cases of patients with pheochromocytoma. Lindstrom et al. reported the use of clevidipine as the sole antihypertensive agent in two adult patients with pheochromocytoma undergoing elective open adrenalectomy, a surgery characterized by a high risk of acute intraoperative hypertension (18). A 4 mg·h^−1^ clevidipine infusion was started immediately after induction of general anesthesia, and the dose administered was increased over the tumor manipulation period by adding additional boluses of 1 mg as needed. This approach ensured an optimal achievement of targeted blood pressure during surgery, although a dose-related increase in HR was observed, requiring the administration of esmolol. Similarly, the role of clevidipine in the management of pheochromocytoma manipulation-induced hypertension was also investigated by Luis-García et al. The authors reported that blood pressure control was obtained within 5 min after the start of an 8 mg·h^−1^ clevidipine infusion. After the re-positioning of the patient, a new hypertensive peak was observed and optimally treated with a 2 mg·h^−1^ clevidipine infusion (19).

#### 3.2.2 Maxillo-facial surgery

In the setting of maxillo-facial surgery, insightful perspectives on the use of clevidipine can be derived from a case report by Kline et al. The patient gradually developed intraoperative hypertension, which was eventually ascribed to an undiagnosed pheochromocytoma. The hypertensive state was refractory to escalating doses of esmolol, labetalol, hydralazine, and nitroglycerin. Blood pressure values were eventually controlled with a clevidipine infusion (up to 5 mg·min^−1^), with the immediate resolution of the hypertensive state (20).

#### 3.2.3 Interventional radiology procedures

In the neuroradiology setting, tight control of blood pressure is of paramount importance, particularly when procedures such as the coiling of cerebral artery aneurysms are performed (21).

In this regard, Meyer et al. reported a case series describing the management of three patients undergoing coiling of cerebral aneurysms under general anesthesia in the interventional radiology laboratory (21). In two patients, clevidipine infusion was started upon emergence from general anesthesia, while in one other patient clevidipine was used intraoperatively. In all patients, clevidipine (used with a dosage up to 8 mg·h^−1^) was effective in controlling blood pressure within the desired, clinician-determined, value within a maximum of 5–7 min. All patients were discharged without complications.

#### 3.2.4 Neurosurgery

Three studies investigated the role of clevidipine in the neurosurgical setting. All of them were published between 2010 and 2020 and were conducted in the United States.

In a study published in 2010, Bekker et al. explored the role of clevidipine as an antihypertensive agent in the perioperative period for neurosurgical patients, to assess the proportion of patients for whom clevidipine could be used without additional antihypertensive drugs to control blood pressure. A total of 22 patients were enrolled in the study. All patients underwent general anesthesia. One patient did not require antihypertensive therapy; 17 patients (81% of the total) received clevidipine alone; one patient received clevidipine in the PACU only. Clevidipine was titrated according to institutional protocol, to obtain SBP < 130 mmHg (starting from 5 to 10 mg·h^−1^ and up to 50 mg·h^−1^). Clevidipine alone was effective in controlling blood pressure in 17 of 21 patients (81%); three patients required labetalol and hydralazine. Target SBP was obtained within 5 min in 14 of 28 episodes (50%) and within 15 min in 22 of 28 episodes (78.6%). Notably, blood pressure elevations during emergence from general anesthesia required higher doses of clevidipine (5.7 ± 5.8 mg), in comparison with hypertensive episodes during both anesthesia induction (1.4 ± 0.7 mg) and maintenance (2.9 ± 1.2 mg). Sixteen patients required metoprolol, although it was chosen as first line treatment in case of occurrence of tachycardia and not necessarily due to uncontrolled blood pressure. Two hypotensive episodes requiring treatment occurred after clevidipine administration. Both the episodes were described as mild hypotension (SBP of 82 and 80 mmHg) and were rapidly resolved with the discontinuation of clevidipine and subsequent administration of either ephedrine or phenylephrine (22).

A subsequent study by Borrell-Vega et al. assessed the efficacy of clevidipine to control blood pressure in the neurosurgical population, after failure of the first line treatment (i.e., nicardipine). Twelve neurosurgical patients were enrolled in the study. The switch from nicardipine to clevidipine occurred based on the clinician’s judgment and the need for additional antihypertensive therapy despite maximal doses of nicardipine. The median number of events requiring an adjustment of the dose was 20.5 vs. 17 during the administration of nicardipine and clevidipine, respectively (*p* = 0.534). The median percentage of time spent at target SBP was 76.2% (IQR: 51.0–93.3) during the administration of nicardipine and 93.4% (IQR: 73–100) during the administration of clevidipine (*p* = 0.123). Moreover, the median percentage of time spent with tachycardia (HR > 100) was 13.1 and 2.2% during the administration of nicardipine and clevidipine, respectively (*p* = 0.250) (23).

Notwithstanding these promising aspects of clevidipine as an antihypertensive agent, this drug is not devoid of adverse effects. A case report by Short et al. described the occurrence of extreme hypoxia induced by clevidipine in a neurosurgical patient. A 16-year-old boy was admitted to the neurosurgical ICU following removal of a thalamic/basal ganglia arteriovenous malformation, complicated by intracerebral bleeding. A clevidipine infusion was started and titrated to 10 mg·h^−1^ upon the patient’s admission to the ICU. 15 h after the beginning, the infusion rate was adjusted to 16 mg·h^−1^ (the maximum dosage recommended by the manufacturer). The patient’s oxygen saturation began to drop concurrently with the increase in the clevidipine dose, and it prompted an increase in the inspired fraction of oxygen (FiO_2_) and positive end-expiratory pressure (PEEP). Despite adjustments in the ventilator settings, the oxygen saturation continued to rapidly decrease. A chest radiograph showed no abnormalities, while further diagnostic workup reduced the clinical concern of massive pulmonary embolism. The consultation with a critical care pharmacist suggested that clevidipine may have been the potential cause of hypoxemia, secondary to clevidipine-induced pulmonary vasodilation and shunting. Within 1 h from the discontinuation of clevidipine, saturation of peripheral oxygen (SpO_2_) quickly improved and partial pressure of oxygen (PaO_2_) rapidly normalized (24).

#### 3.2.5 Vascular surgery

In 1997, Huraux et al. investigated the properties of clevidipine on human IMA musculature. The authors surgically obtained IMA segments and precontracted them with an analog of thromboxane. Then, acetylcholine and nitroglycerin were added to investigate the endothelial function. In the IMA samples with endothelium, acetylcholine did not completely reverse thromboxane-mediated contraction, while both clevidipine and nitroglycerine were effective in obtaining a full reversal. The response to clevidipine remained consistent in both preparations, with and without intact endothelium, confirming the endothelium-independent antihypertensive properties of clevidipine (25).

Another setting where blood pressure control is of paramount importance is the case of aneurysmectomy as described in a case report by Pascual et al. After induction of general anesthesia for the surgical excision of a pseudo-aneurysm, the patient developed a marked hypertensive state, refractory to urapidil boluses (40 mg intravenous boluses repeated twice). Thus, a clevidipine infusion was started and escalated from 2 to 6 mg·min^−1^, allowing for an effective management of blood pressure. The authors did not observe modifications in heart rate. Clevidipine was then gradually de-escalated in the ICU and eventually stopped 10 h after its initiation (26).

## 4 Discussion

### 4.1 Key findings

This review highlights a favorable efficacy and safety profile of clevidipine for the perioperative management of hypertension—especially in cardiac surgery—when a tight control of blood pressure is of importance to avoid adverse patient outcome. Hypertension is, indeed, one of the most common perioperative abnormalities, and was found to occur in up to 80% of cardiac surgery patients and 25% of patients undergoing noncardiac surgery (27). To date, numerous antihypertensive drugs are available, and a recent survey highlighted that the main features leading to the antihypertensive agent choice by the attending anesthesiologist are the drug’s ease-of-use and the clinician experience with that particular drug, while the most appreciated feature was reported to be the titratable control (28).

In this regard, clevidipine may be a valuable addition to other commonly used antihypertensive drugs, and demonstrated a favorable efficacy, safety profile and ease-of-use in trials that compared it to SNP, nicardipine and nitroglycerine (11).

Moreover, a relevant feature arising from a subsequent analysis of the ECLIPSE trial is the predictable effect of clevidipine on blood pressure values of different patients, throughout the whole perioperative period (12). This result was inferred from investigations conducted in the cardiac surgery setting, but it may be reasonable to assume that it is applicable to almost any other perioperative setting.

Yet, clevidipine is not completely devoid of drawbacks, and one of the most common adverse events reported in literature is tachycardia, occasionally necessitating the administration of additional medications (e.g., concomitant β-blockers) (10, 13, 18, 21, 23). Moreover, anecdotal evidence reported the case of severe hypoxemia (presumably due to pulmonary vasodilation and shunt mechanism) following a prolonged clevidipine infusion. However, the ultra-short half-life of clevidipine allowed for a prompt resolution of the clinical picture following discontinuation of the intra infusion (24). As such, these aspects must be carefully considered and require both further research endeavors and additional thorough evaluations of patient-specific characteristics before the administration of this drug.

### 4.2 Relationship with previous literature

This review summarized the current available evidence regarding the use of clevidipine in the perioperative context, thus providing useful insights about the potential applications of this novel antihypertensive drug. To date, this review can only be considered as the best recent evidence-based practice.

In cardiac surgery, clevidipine has been extensively investigated and compared to other common antihypertensive drugs. Specifically, the ECLIPSE trial and its subsequent analysis showed that clevidipine is more effective in obtaining target blood pressure values than nitroglycerine, SNP and, in cases when the desired range of target blood pressure is narrowed, nicardipine (11, 12). Remarkably, blood pressure values obtained with clevidipine were similar in different patients, and its use allowed for a lesser degree of variation than the one observed with nicardipine and SNP. Moreover, in this setting, clevidipine showed superiority versus placebo in two distinct randomized controlled trials (13, 14). Additionally, while mortality rates in the ECLIPSE trial did not differ between clevidipine, nitroglycerine, and nicardipine groups, these were remarkably higher in the SNP group (11).

In the setting of general surgery, clevidipine proved to be effective in the management of pheochromocytoma-related hypertension, even when resistant to escalating doses of other commonly used antihypertensive drugs, allowing for a rapid and effective control of blood pressure (18–20).

Clevidipine use was also vastly tested in the neurosurgical population, for both interventional radiology procedures, where a case series 23 proved clevidipine to be effective in managing the hemodynamics of patients undergoing cerebral artery aneurysm coiling, and neurosurgery, a setting where clevidipine showed promising results, even after the failure of first line treatment with nicardipine (22, 23).

Across all of these settings, the predictable effects of clevidipine and the lesser degree of blood pression excursion both above and below the targeted blood pressure range of values are of remarkable value as perioperative hypotension is known to be associated with myocardial injury, acute kidney injury, and death (29, 30). In this regard, the introduction of an antihypertensive agent that shows a minimal risk of hypotension holds promising venues to a more effective and safe perioperative blood pressure management.

### 4.3 Implications for clinical practice and future research

The comprehensive review of data retrieved from literature supports the use of clevidipine in daily clinical perioperative practice, highlighting both its peculiar efficacy and ease-of-use.

In those settings where close control of blood pressure is crucial, clevidipine is an optimal drug, due to its rapid onset, vascular selectivity, and extremely short half-life (9).

Of the retrieved articles, only one showed potential safety concerns regarding extreme hypoxemia following the administration of clevidipine in a neurosurgical patient. The hypoxemia was likely attributed to the vasodilatory properties of clevidipine and its effect on pulmonary vessels, which is capable of inducing pulmonary shunt by overcoming the physiological pulmonary hypoxic vasoconstriction. This side effect has been previously reported with other dihydropyridine calcium channel blocker. A pharmacovigilance study of the VigiBase database revealed that, among dihydropyridine calcium channel inhibitors, iv clevidipine and nicardipine were significantly associated with hypoxia (31). However, the ultra-short half-life of clevidipine mitigated the potential adverse outcomes of such scenario, that rapidly resolved with the discontinuation of the clevidipine infusion (24). Nevertheless, when a concern for hypoxia is present, other antihypertensive drugs (e.g., urapidil, labetalol) may represent a valid alternative, and may be administered on a case-by-case basis.

In a number of the retrieved studies, tachycardia was reported in patients treated with clevidipine, generally occurring shortly after initiation of the infusion (10, 13, 18, 21, 22), while in other studies no such effect was observed (5, 17, 26). This could be explained as a correlation rather than a causation, given the fact that several stimuli during the perioperative period could prompt changes in the HR; nonetheless, this aspect is worthy of further investigations, especially in the context of cardiac patients where tachycardia is a particularly undesirable side effect.

Still, what essentially emerges from the reviewed literature is that clevidipine appears as an attractive antihypertensive drug, appropriate for use in the perioperative period, allowing for an accurate and timely control of blood pressure within a physician-determined range, with great ease-of-handling and swift titration of the therapeutic effect.

Nevertheless, half of the retrieved studies (which account for most of the included patient population) focused on the application of clevidipine in the context of cardiac anesthesia. Further research endeavors aiming at both clarifying the potential advantages and/or drawbacks of clevidipine in different populations (e.g., frail and elderly patients) and across various surgical settings, as well as to better evaluate the pharmacokinetic and pharmacodynamic properties of this agent in different ethnical groups and across different age ranges, are needed.

### 4.4 Strengths and limitations

To the best of the authors’ knowledge, this is the most up-to-date review summarizing the key features of clevidipine across several perioperative settings. Methodological strengths of this review include a well-defined research question and a focused analysis of a specific population (i.e., patients undergoing cardiac and noncardiac surgical procedures who developed perioperative hypertension). Moreover, clevidipine has been extensively compared to the most commonly used antihypertensive agents (e.g., nicardipine, SNP, nitroglycerin), providing a valuable synthesis of its properties to guide the daily anesthetic management of perioperative hypertension. Furthermore, most of the included studies were assessed to be at a “low risk of bias”.

However, this study suffers some limitations. Firstly, while this review encompasses studies characterized as high level of evidence (4 of the retrieved studies were RCTs), several additional findings were extrapolated from observational studies, as well as case reports and case series (i.e., studies with a lover strength of evidence). Secondly, most of the included studies were published more than 10 years ago. Furthermore, the majority of the studies were conducted in the cardiac surgery setting, with high-quality evidence still lacking for other noncardiac surgical settings, and data in the noncardiac surgical settings derived from a limited number of patients. Additionally, the heterogeneity in the retrieved studies—in terms of clinical setting, patient populations, comparators (or lack of comparators)—limited the ability to provide a formal data synthesis. Moreover, drug-related costs are often decisive in the widespread use of a novel agent in contemporary healthcare systems. In this regard, while clevidipine proved be to more cost-effective than SNP, it resulted more expensive than nicardipine (17, 32). While a cost analysis falls beyond the scope of the present review, it is imperative for further research endeavors to thoroughly address this important aspect. Lastly, a number of different doses and timing of clevidipine administration were reported, thus potentially impairing—at least in part—the generalizability and comparability among studies of the findings of this review.

## 5 Conclusion and future perspectives

This present systematic review aimed to elucidate on the role of clevidipine in the management of perioperative hypertension. This novel drug, with its rapid onset and offset, predictable dose–response, and ease of titration to the desired effect regardless of patient weight, has demonstrated promising properties for perioperative blood pressure management.

However, several gaps in our understanding of this novel molecule still need clarification, such as the impact of genetics and ethnicity on its metabolism, as well as its use in frail and elderly patients, particularly in the noncardiac surgery setting. Therefore, further research is warranted to better investigate and define the potential role of clevidipine among the available perioperative antihypertensive agents.

## Data Availability

The original contributions presented in the study are included in the article/Supplementary material, further inquiries can be directed to the corresponding author.

## Footnotes

^1^Accessible online at: https://www.riskofbias.info.
